# The multifaceted role of phosphodiesterase 4 in tumor: from tumorigenesis to immunotherapy

**DOI:** 10.3389/fimmu.2025.1528932

**Published:** 2025-03-10

**Authors:** Huili-li Ren, Shao-hui Zhang, Pei-yuan Li

**Affiliations:** ^1^ Department of Pharmacy, Traditional Chinese and Western Medicine Hospital of Wuhan, Tongji Medical College, Huazhong University of Science and Technology, Wuhan, China; ^2^ Division of Gastroenterology, Tongji Hospital, Tongji Medical College, Huazhong University of Science and Technology, Wuhan, China; ^3^ Department of Gastroenterology, Wenchang People’s Hospital, Wenchang, Hainan, China

**Keywords:** phosphodiesterase 4 (PDE4), tumorigenesis, drug resistance, tumor immunity, PDE4 inhibitors

## Abstract

Phosphodiesterase 4 (PDE4) is an enzyme that specifically hydrolyzes the second messenger cAMP and has a critical role in the regulation of a variety of cellular functions. In recent years, PDE4 has attracted great interest in cancer research, and its role in tumorigenesis and development has been gradually elucidated. Research indicates that abnormal expression or heightened activity of PDE4 is associated with the initiation and progression of multiple cancers, including lung, colorectal, and hematological cancers, by facilitating cell proliferation, migration, invasion, and anti-apoptosis. Moreover, PDE4 also influences the tumor immune microenvironment, significantly immune evasion by suppressing anti-tumor immune responses, reducing T-cell activation, and promoting the polarization of tumor-associated macrophages toward a pro-tumorigenic phenotype. However, the PDE4 family may have both oncogenic and tumor-suppressive effects, which could depend on the specific type and grade of the tumor. PDE4 inhibitors have garnered substantial interest as potential anti-cancer therapeutics, directly inhibiting tumor cell growth and restoring immune surveillance capabilities to enhance the clearance of tumor cells. Several PDE4 inhibitors are currently under investigation with the aim of exploring their potential in cancer therapy, particularly in combination strategies with immune checkpoint inhibitors, to improve therapeutic efficacy and mitigate the side effects of conventional chemotherapy. This review provides an overview of PDE4 in tumorigenesis, drug resistance, immunotherapy, and the anti-tumor actions of its inhibitors, intending to guide the exploration of PDE4 as a new target in tumor therapy.

## 1 Introduction

Phosphodiesterases (PDEs) are a class of enzymes that catalyze the cyclic adenosine monophosphate (cAMP) and cyclic guanosine monophosphate (cGMP) (1). The PDE superfamily is divided into 11 families (PDE1-PDE11), classified by structure and enzymatic properties (2). Serving as a secondary messenger, cAMP plays a crucial role in regulating cell proliferation (3–5), differentiation (6, 7), apoptosis (8–11), and immune responses (9, 12, 13), by influencing the development of both physiological and pathological processes. The PDE4 family is arguably the most studied of all the PDE families. However, as an important PDE subtype that catalyzes the hydrolysis of cAMP, PDE4 has gradually attracted researchers’ attention for its role in modulating a variety of pathological processes. The PDE4 family comprises several subtypes, notably including PDE4A, PDE4B, PDE4C, and PDE4D. These subtypes are respectively located on different chromosomes: PDE4A at 19p13.2, PDE4B at 1p31, PDE4C at 19p13.11, and PDE4D at 5q12 (14). Each subtype can be divided into long, short, and ultrashort forms based on molecular weight. This diversity underpins the specific functions of PDE4 in various cell types and tissues (15). These subtypes exhibit differential cellular distributions and carry out unique roles in both physiological and pathophysiological processes. For example, PDE4B is highly expressed in neutrophils and other immune cells (16–18), closely related to inflammatory responses. Whereas PDE4D is abundant in the heart (19, 20) and brain (21–23), contributing to cardiovascular function and memory formation. The specific expression patterns and functions of PDE4 subtypes offer possibilities for developing targeted therapies for specific diseases.

Growing evidence highlights the complex relationships between PDE4 and various aspects of cancer. By regulating cAMP levels, PDE4 subfamilies were involved in tumorigenesis (24–28), metastasis (29–31), drug resistance (32, 33), and immune microenvironment (34–37). However, due to the PDE4 family having multiple subtypes and the heterogeneity of tumor cells, the role of PDE4 in tumor progression is also complex and varied. For instance, PDE4A appears to act as an oncogene in hepatocellular carcinoma cell line Huh7 (38), whereas it exhibits tumor-suppressive effects in HepG2 and BEL7402 cells (39). Thus, the PDE4/cAMP signaling pathway has emerged as a promising but extremely challenging target for drug development due to its strong association with tumor progression.

Advancements in understanding the structure and function of PDE4, have propelled the development of small molecule inhibitors targeting PDE4. Several PDE4-selective inhibitors have successfully reached the market, targeting diseases such as psoriasis, atopic dermatitis, chronic obstructive pulmonary disease (COPD), asthma and alleviating pain caused by smooth muscle spasm (**Table 1**). More recent work is examining PDE4 in the context of cancer, including B-cell lymphomas (40), triple negative breast cancer (41), and colorectal cancer (42). These studies offer promising insights into the potential of PDE4 inhibitors to target tumors.

**Table 1:** Marketed PDE4 inhibitors.

Compound	Indication
Rolipram	Application for asthma treatment (43)
Roflumilast	Approved in the European Union in 2010 for patients with COPD and in the United States in 2022 for treating plaque psoriasis (44–46)
Cilomilast	Chronic Obstructive Pulmonary Disease (47, 48)
Apremilast	Psoriatic arthritis (49)
Crisaborole	Moderate atopic dermatitis
Drotaverine	Functional bowel disorders and alleviating pain caused by smooth muscle spasm
Ibudilast	Rare childhood disease, Krabbe disease, bronchial asthma

This review aims to provide a comprehensive overview of the latest progress in understanding the relationship between PDE4 and cancer, examining its molecular mechanisms in tumorigenesis, drug resistance, and immunotherapy, summarizing the status and challenges of PDE4 inhibitors in tumor treatment, and prospecting the future research directions.

## 2 PDE4 and tumorigenesis

**Figure 1:**
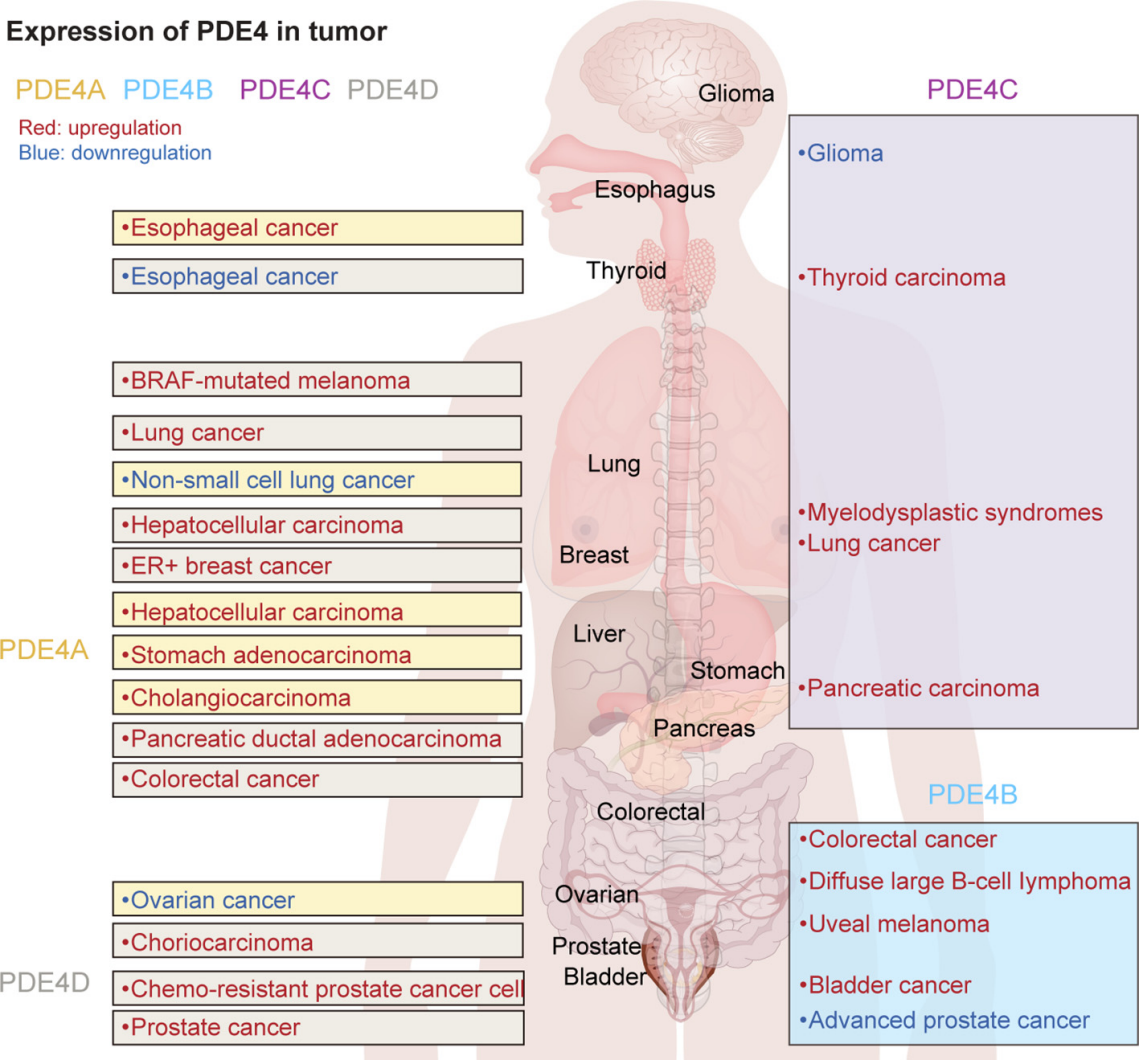
The expression of PDE4 subtypes in various tumors.

Various patterns of expression and localization within cells and tissues significantly influence the specific roles and functions of PDE4 subfamilies and isoforms (15). Additionally, researchers have found that the expression of different PDE4 subtypes in tumor cells varies significantly (**Figure 1**). It suggests that PDE4 could play either a promotional or inhibitory role in the development and progression of tumors, depending on the type of disease and its stage of advancement.

### 2.1 PDE4A and cancer

PDE4A is one of the PDE4 subfamilies. Malfunction or altered regulation of PDE4A has been connected to a multitude of diseases ranging from neurological disorders like Alzheimer’s and Parkinson’s diseases to psychiatric conditions such as depression and anxiety (50, 51). Furthermore, it also implicated in chronic inflammatory diseases including asthma and COPD (52). Recently, accumulating researches suggest a potential role for PDE4A in cancer development, highlighting its complex influence across physiological process (38). PDE4A has been found to be expressed in medulloblastomas, glioblastomas, oligodendrogliomas, ependymomas, and meningiomas (53), and may be a new target for treating brain tumors. Additionally, PDE4 inhibitor not only directly inhibits the proliferation of glioma cells (54), but also combination with first-line therapy for malignant gliomas (such as temozolomide), enhances the survival rate of mice with intracranial implants of U87 glioblastoma cells (53).

**Figure 2:**
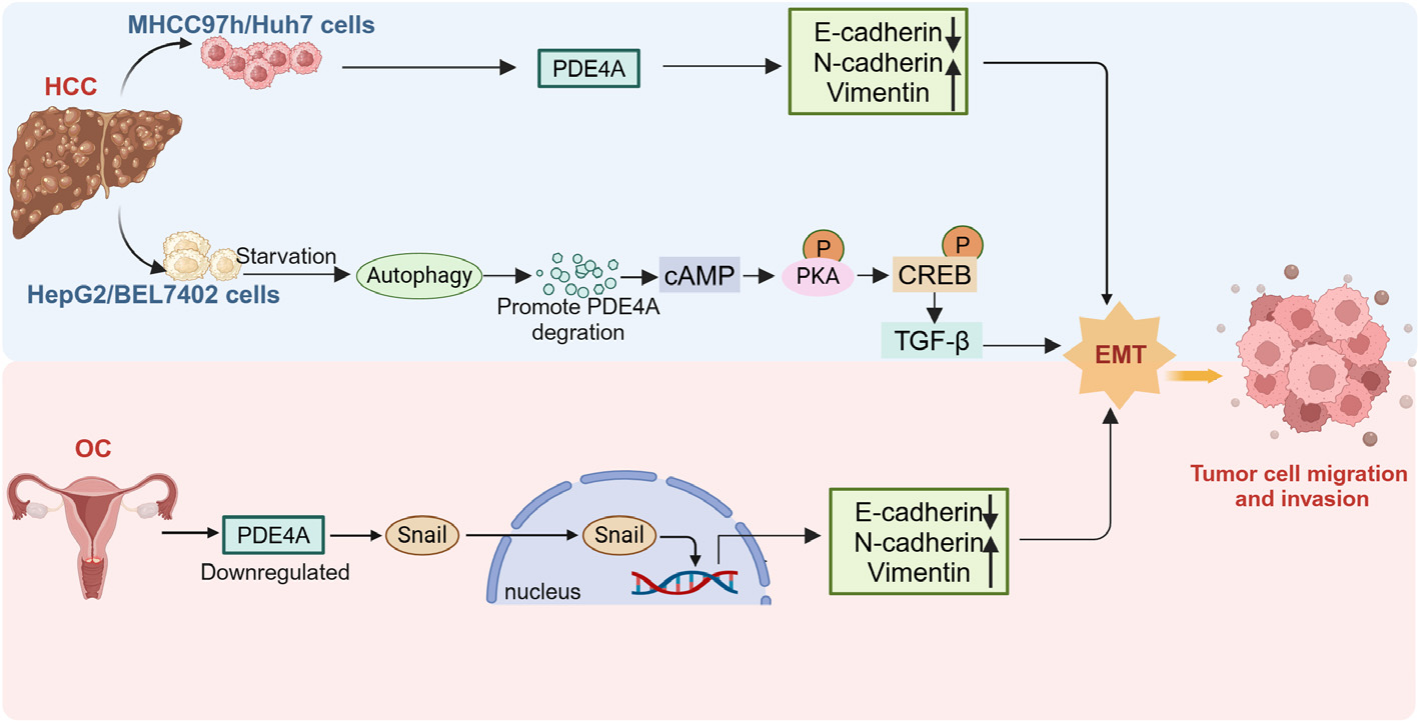
The different role of PDE4A in tumor EMT. The expression of PDE4A is upregulated in MHCC97H and Huh7 cell, and promote the EMT. PDE4A is degraded by starvation induced autophagy in HepG2 cells, thus promoting EMT conversion. Downregulation of PDE4A expression in ovarian cancer induce EMT and nuclear translocation of Snail. HCC, hepatocellular carcinoma; OC, ovarian cancer; EMT, epithelial-mesenchymal transition (This figure was created with Biorender.com).

However, PDE4A may be protumorigenic or antitumorigenic, depending on the type of tumor or the type of tumor cells. It is reported that most patients with hepatocellular carcinoma (HCC) show elevated PDE4A expression in tumor tissues relative to corresponding adjacent liver tissues (38). Furthermore, increased PDE4A expression in tumor tissues is positively correlated with hepatitis B virus (HBV) infection, liver cirrhosis, higher levels of serum alpha-fetoprotein (AFP), advanced TNM staging, the presence of vascular emboli, intrahepatic metastasis, and portal vein tumor thrombus (PVTT) (38). Knockout of PDE4A in Huh7 cells inhibited cell migration and epithelial-mesenchymal transition (EMT) (**Figure 2**). Nevertheless, research also shows that PDE4A protects against EMT in HepG2 and BEL7402 cells (39). Autophagy triggers increased TGF-β1 expression and induces EMT in HepG2 and BEL7402 cells which occurs through the cAMP/PKA/CREB signaling pathway. Notably, this activation is facilitated by autophagy-mediated degradation of PDE4A (39) (**Figure 2**). The phenomenon of PDE4A exerting differential impacts on EMT across various HCC cells might be ground in the heterogeneous characteristics of the cells. This highlights the necessity for in-depth investigation into the precise mechanisms by which PDE4A modulates EMT in HCC.

PDE4A is significantly upregulated in several hematologic tumors (55), stomach adenocarcinoma, and cholangiocarcinoma, and is associated with cell proliferation (56). Conversely, although PDE4A expression is also elevated in estrogen receptor-positive and progesterone receptor-positive breast cancer patients, patients with high PDE4A expression have longer progression-free survival (PFS) and overall survival (OS) (57). Similarly, low expression of PDE4A has been proved to promote the progression of ovarian cancer (OC) by inducing snail nuclear translocation (27) (**Figure 2**). These findings indicate that PDE4A may exert different roles depending on the tumor type.

During the process of tumorigenesis, multiple factors were reported to modulate the expression or activity of PDE4A. In non-small cell lung cancer (NSCLC) tissues, there is a reduction in PDE4A protein expression, while mRNA level exhibits an inverse trend (56). This indicates the potential presence of post-translational modifications of PDE4A in NSCLC. Hypoxia is a consequence of excessive oxygen consumption by tumor cells and insufficient oxygen supply owing to a compromised vasculature. In A549 adenocarcinoma cells, the expression of PDE4A and PDE4D increase under hypoxic conditions and enhance HIF signaling, which promotes the proliferation of tumor cells (58). Chewing betel nut is closely related to oral submucous fibrosis, considered a precancerous condition. Arecoline enhances TGF-β-induced buccal mucosal fibroblast (BMF) activation by modulating the activity of PDE4A without altering the expression of PDE4A (54). PDE4A showed a critical role in the progression of esophageal squamous cell carcinoma (ESCC) through its regulation by lncRNA HCP5. This interaction also activates the PI3K/AKT/mTOR signaling pathway and provided insights that may enhance therapeutic strategies for ESCC (59). In summary, PDE4A not only acts directly on tumor cells to promote their growth but also influences the tumor microenvironment. Further research is needed in the future to clearly define the specific role of PDE4A in tumor development and its potential therapeutic implications.

### 2.2 PDE4B and cancer

The PDE4B gene resides on chromosome 1p31, with enlightening single nucleotide polymorphisms (SNPs) clustering in the region encoding PDE4B predominantly situated at the 5′-terminals (50). Furthermore, the PDE4B gene also encodes PDE4B monomers known as PDE4B2 and PDE4B5 (71). Altered expression of PDE4B is involved in a wide range of disease progression, including Parkinson’s disease (60), asthma (61), hematologic malignancies (62), and other cancer diseases (24, 63).

Melanoma is a highly malignant skin tumor with a significant propensity for metastasis. Given its propensity for metastasis, the manner and site of melanoma spread significantly influence patient prognoses (64). PDE4B was identified as a prognostic gene in metastatic melanoma by multiple bioinformatics approaches analysis (65). In addition, the expression of PDE4B was also associated with the prognosis of uveal melanoma (66). Silencing PDE4B or PDE4D significantly reduced PDE4 activity in melanoma cells, whereas PDE4A had no such effect (67), suggesting that PDE4B and PDE4D play crucial roles in regulating PDE4 activity in melanoma. Further investigation revealed that PDE4B2 is required for the oncogenic Ras-driven melanoma (67).

Patients with advanced urinary bladder cancer (UBC) exhibit increased levels of PDE4B mRNA (68) which predicts poor prognosis in UBC Patients. Mechanistically, PDE4B is negatively regulated by chromobox protein homolog 7 (CBX7) in a PRC1-dependent manner in UBC cells. However, the role of PDE4B in prostate cancer appears to differ from that in UBC. Alterations in PDE sequences are closely associated with the development of prostate cancer. In the early stages, the proliferation of prostate cancer cells relies on androgens, and androgen deprivation therapy can suppress the growth of prostate cancer cells (69, 70). However, most prostate cancers progress to a castration-resistant phenotype, where treatments based on androgen docetaxel often prove minimally effective. It was found that oxidative stress inhibits PDE4B expression during this process, which activates PKA and promotes cell proliferation (71). This study demonstrates that PDE4B may perform a tumor suppressor role in advanced prostate cancer, but further studies are needed to confirm this role.

**Figure 3:**
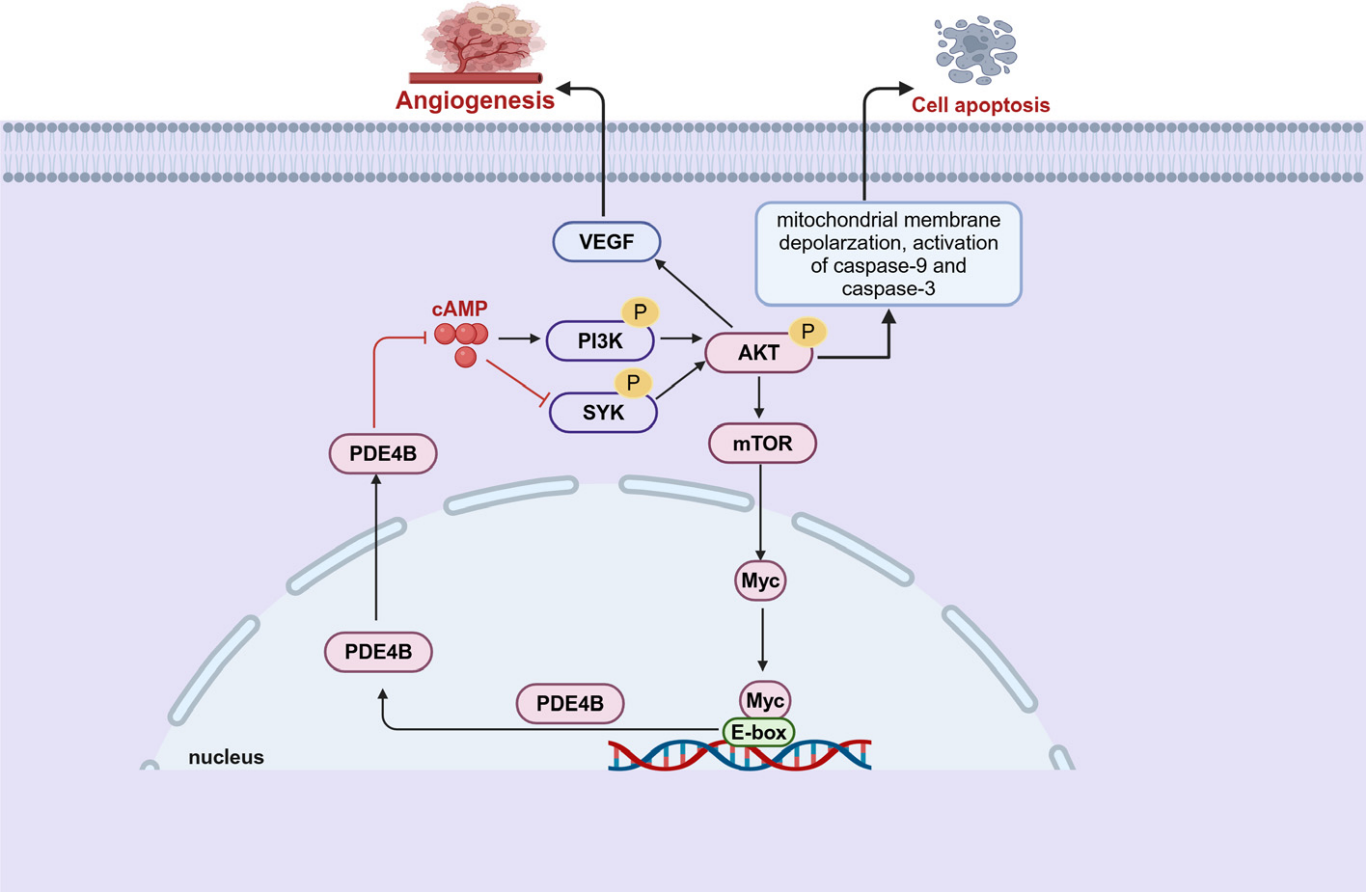
PDE4B regulates tumor angiogenesis and apoptosis through multiple pathways in B-cell lymphoma. There is a pre-feedback path between Myc and PDE4B. PDE4B regulates Myc expression through the SYK/AKT/mTOR pathway. Meanwhile, Myc regulates the expression of PDE4B by directly binding to the E-box of the PDE4B promoter (This figure was created with Biorender.com).

Initial evidence implicating a connection between PDE4B and B-cell lymphoma emerged from comprehensive genomic profiling analyses (72). These studies indicate that PDE4B expression is upregulated in patients with diffuse large B-cell lymphoma (DLBCL) who have developed resistance to chemotherapy (72). The following studies have demonstrated that cAMP promoted DLBCL cells apoptosis by inhibiting the PI3K/AKT pathway. Notably, this effect is suppressed when cAMP is degraded via the upregulated of PDE4B (73) (**Figure 3**). Furthermore, the cAMP-PDE4B axis is also linked to the tumor microenvironment of DLBCL (74, 75). Angiogenesis plays a critical role in the prognosis of DLBCL, and there is evidence that PDE4B influence this process. Mechanistically, PDE4B enhances hypoxia-induced VEGF production by catalyzing cAMP hydrolysis, which regulates the tumor microenvironment and promotes angiogenesis (74) (**Figure 3**). Extensive research indicates that B-cell lymphomas with enhanced Myc expression are associated with an aggressive phenotype and poor prognosis (76, 77). While, there may exist a Myc-PDE4B positive feedback loop which cooperatively enhance the proliferation of DLBCL cells (78) (**Figure 3**). In cells with high PDE4B expression, PDE4 inhibitors augment the JQ1-mediated cell death, whereas this synergistic action is not observed in cells with low PDE4B expression (78). This indicates that B-cell lymphomas might necessitate stratification into PDE4B-high and PDE4B-low expression groups to facilitate personalized combination therapies incorporating PDE4 inhibitors.

Colorectal cancer (CRC) is regarded as the most commonly diagnosed malignancy within the gastrointestinal tract. It lacks homogeneity and can be categorized into various subtypes characterized by specific molecular and morphological changes. Research has revealed that PDE4B is conserved across three mammalian species—mouse, rat, and human—and exhibits differences between colorectal adenomas and normal tissue (79). Loss of PDE4B function in the ApcMin/+ mouse, a spontaneous pre-cancerous colonic lesion model mouse, results in a significant increase in the number of colonic adenomas (79). This finding implies that PDE4B can prevent ApcMin-induced adenoma formation and protect against the early stages of colon cancer in the mouse. However, the study revealed that while the expression of PDE4B is elevated in patients with advanced colorectal cancer, its functional activity is conversely diminished. Similarly, another research showed the highest PDE4B expression in the control tissue and Dukes’ A stage (80). However, in patients with advanced colon cancer, PDE4B may become inactivated through epigenetic suppression (80). PDE4B is also excessively expressed as a dysfunctional protein in non-neoplastic appearing colonic mucosa from patients with colorectal neoplasia (81). Oncogenic KRAS specifically upregulates PDE4B2 in a three-dimensional (3-D) colonic crypt model (82). Suppression of PDE4 catalytic activity can induce epithelial cell polarity and apoptosis within the luminal space in CRC (82).

### 2.3 PDE4C and cancer

In contrast with other PDE4 subtypes, current evidence indicates that PDE4C gene expression yields exclusively long-form enzyme isoforms. Consequently, every PDE4C enzyme features a distinct N-terminal segment, paired with two invariant regulatory segments (designated UCR1 and UCR2), a conserved catalytic region, and a C-terminal tail characteristic to its subfamily, adhering to the overarching modular architecture described for PDE4 enzymes (83). Mounting evidences suggest that the PDE4C is associated with a variety of diseases, including cancer (83). The Human Protein Atlas (HPA) provides comprehensive and significant insights into the expression of PDE4C across different types of cancer (84). As described in HPA, PDE4C exhibited harmful effects in five types of cancer: renal carcinoma, glioblastoma, pancreatic carcinoma, melanoma, and breast cancer (84). Instead, it shows a protective role in four cancers: head and neck carcinoma, urothelial carcinoma, gastric carcinoma, and cervical cancer (84). Compared to other PDE4 subtypes, PDE4C tends to exhibit lower expression levels in majority of tumors. However, PDE4C emerged as the sole prominent member of the PDE4 family in glioma and pancreatic cancer specifically. Notably, elevated PDE4C expression is strongly associated with lower survival and poorer treatment outcomes in a variety of malignant tumors, such as myelodysplastic syndromes (MDS), pancreatic cancer, and lung cancer (55, 85, 86).

**Figure 4:**
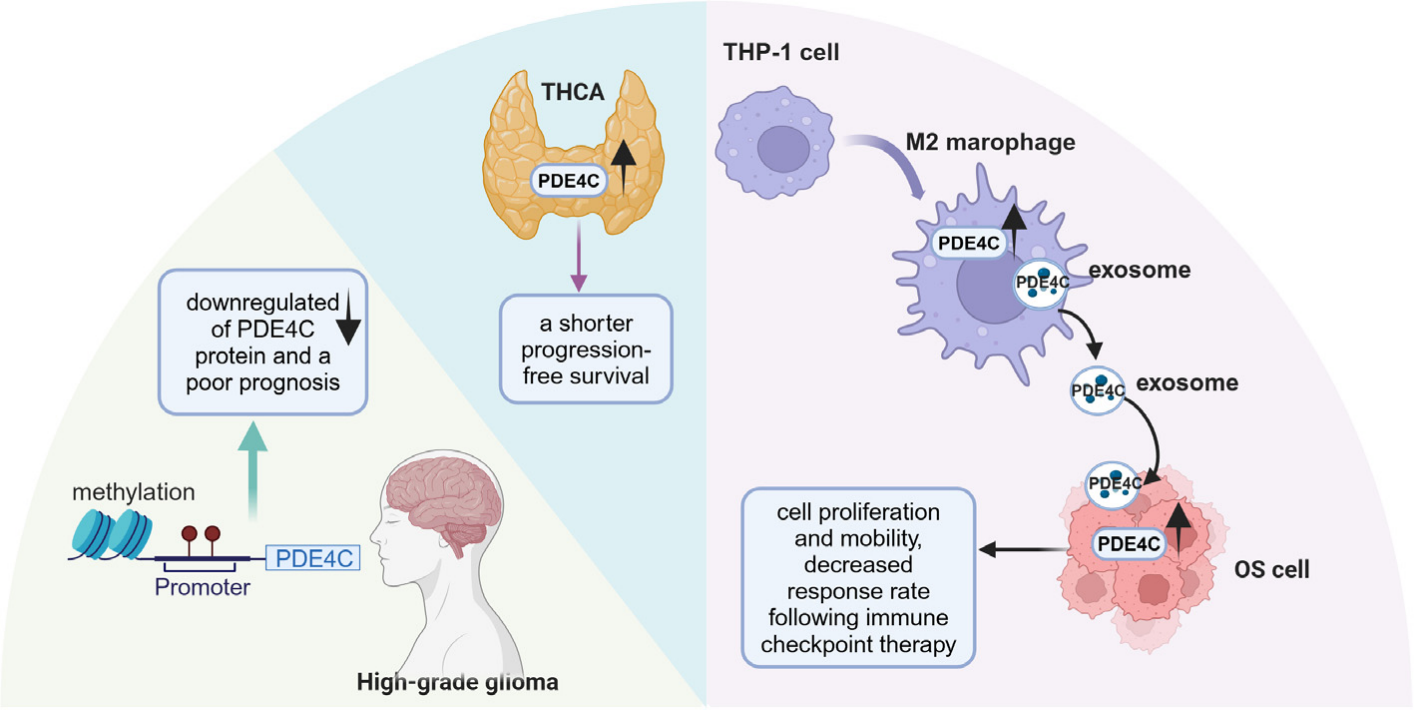
The role of PDE4C in different tumor progression. PDE4C had significant promoter methylation and lower expression in high-grade glioma. PDE4C expression was elevated in THCA and is associated with poor prognosis. The expression of PDE4C was observed to increase during the conversion process of THP-1 cells to M2 macrophage, which transferred the PDE4C mRNA to OS cells through exosome approach. TCHA, thyroid carcinoma; OS, osteosarcoma (This figure was created with Biorender.com).

In patients with thyroid carcinoma (THCA) across various stages, the expression of PDE4A, PDE4B, and PDE4D showed a decrease; conversely, PDE4C expression was notably elevated (87). Furthermore, THCA patients with higher expression levels of PDE4C experienced a shorter progression-free survival than those with lower PDE4C expression (87). Heightened promoter methylation of PDE4C coupled with a reduction in PDE4C protein expression was inversely correlated with overall patient survival rates in brain cancer (88) (**Figure 4**). However, this is inconsistent with the data from the HPA (55). This is potentially due to the HPA’s lack of consideration for the staging of gliomas. Another study has revealed an association in glioma patients between reduced expression of PDE4C and downregulation of apoptotic pathways coupled with upregulation of cell migration pathways by transcriptomic analysis (88). It was found that there was a protective relationship between PDE4C and p53 (88, 89). In cancer cell with p53 mutations, which impede its ability to govern transcription of target genes, transcriptomic profiling consistently pointed to reduced expression of PDE4C (89). Additionally, the p53 regulatory sequence within the PDE4C gene’s promoter zone implies that functional wild-type p53 enhances the expression of the PDE4C gene. These findings indicate a reciprocal regulatory relationship between PDE4C and p53 and disruptions to this protective interplay might foster oncogenesis.

Furthermore, PDE4C has been identified as a crucial intermediary in communication between tumor cells and immune cells. Tumor-associated macrophages (TAMs), including M1 and M2 phenotypes, play a pivotal role in influencing the progression of osteosarcoma (OS) and promoting immunosuppressive environments (90). Increased expression of PDE4C observed in OS tissues, coinciding with heightened levels of M2 macrophages, an unfavorable prognosis, and the presence of metastasis (91) (**Figure 4**). PDE4C secreted by M2 macrophages may promote the proliferation and migratory capabilities of OS cells through elevated collagen production. This suggests that PDE4C might serve as a potential biomarker for predicting prognosis and therapeutic response in OS. In summary, the role of PDE4C in tumors appears to be highly heterogeneous, and further research is warranted to clarify its potential as both a tumor biomarker and a therapeutic target.

### 2.4 PDE4D and cancer

**Figure 5:**
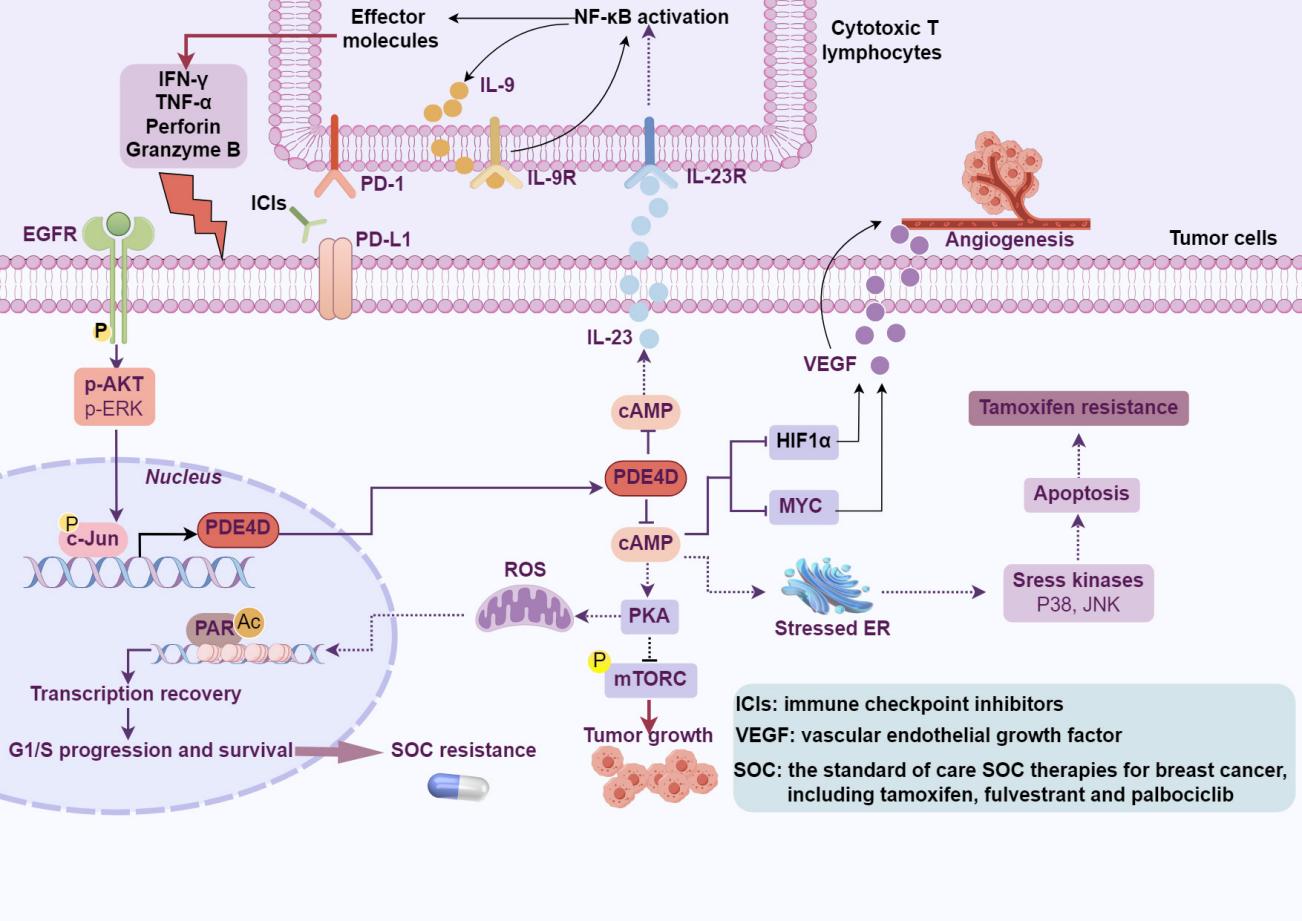
The effects of PDE4D-cAMP signaling on cancer. PDE4D modulates tumor cell growth, angiogenesis, resistance to tamoxifen and SOC treatments, and influences the response to cancer immunotherapy through the hydrolysis of cAMP (This figure was created with Biorender.com).

Multiple protein variants of PDE4D (PDE4D1 through PDE4D11) result from complex arrangements and splicing of PDE4D locus, with nine variants identified in humans and eleven in mice (92). These isoforms generate proteins with diverse lengths, containing or lacking domains vital for their functional roles. They divide into three classifications: extended isoforms such as PDE4D3, PDE4D5-10 in humans and PDE4D11 in mice, compact isoforms represented by PDE4D1, and ultra-compact isoforms observed in mice, specifically PDE4D2 and PDE4D6 (92). Expression patterns of these variants exhibit varying degrees of tissue specificity; notably, PDE4D4 and PDE4D6 are predominantly confined to the brain, while PDE4D8 is found in tissues beyond the confines of the central nervous system (93). Recently, it has been reported that PDE4D gives rise to a notably stable, predominantly localized in the cytoplasm, circular RNA, designated as circPDE4D (94). This circRNA originates from the exon 2 to 5 region of the PDE4D gene through a process of exon circularization (94) and the functional role of circPDE4D remains largely to be determined. Furthermore, extensive studies have been conducted to reveal the role of PDE4D in regulating tumor cell growth, angiogenesis, and responses to immune therapy (**Figure 5**). However, the role of PDE4D in tumor progression is multifaceted, potentially exhibiting both oncogenic and tumor-suppressive activities, which may be associated with the type and stage of the tumor.

Studies found that the PDE4D gene in approximately 20% of prostate tumors (95), and is closely related to tumor progression. In prostates, PDE4D is expressed in both stromal and epithelial compartments, with its hydrolytic activity confined to cytoplasmic compartments (96). However, the role of PDE4D7 in prostate cancer appears distinct from that of PDE4D. PDE4D7 is localized to the submembrane region of prostate cancer cells (97). In addition, the expression of PDE4D7 decreased as the disease advanced from an androgen-sensitive (AS) localized prostate cancer to a castration-resistant form, suggesting its involvement in fostering a more aggressive disease manifestation (97). The differential roles of PDE4D and PDE4D7 in prostate cancer indicate the complex involvement of PDE4D subtypes in the development of prostate cancer, highlighting the need for further research to elucidate the specific functions of each subtype.

Growing evidence has focused on the role of PDE4D in CRC progression, but the results were not consistent. Studies showed that PDE4D acted as an oncogenic protein involved in the development of CRC (28, 98). Inhibition of PDE4D suppresses the malignant properties of DLD-1 cells by inhibiting the AKT/mTOR/Myc signaling pathway (99). Around 5% of CRC cases harbor mutations in the GNAS (guanine nucleotide binding protein, alpha stimulating) gene, resulting in the stimulation of cAMP-mediated signaling pathways and a poor prognosis (27). RNA sequencing results revealed that the most upregulated gene in GNAS-mutated (GNASmt) cells was PDE4D. Notably, the PDE4 inhibitor Ro 20-1724 and PDE4D selective inhibitor GEBR-7b suppressed the proliferation of GNASmt cells while not affecting parental cells. This provides evidence for targeting PDE4D in the treatment of CRC with GNAS mutations. However, one study also found that the PDE4D mRNA was down-regulated in CRC compared with normal colon as revealed by oncomine data-mining analysis and knockdown of PDE4D increase the cell proliferation (100). Some studies suggest that a hallmark of disease progression is the overall downregulation of the long isoforms of PDE4D, whereas the short isoforms (PDE4D1/2) appear to be relatively unaffected (101). Thus, the data indicated that compared to benign tissue, the long isoforms of PDE4D may be altered in primary human CRC.

For metastasis of choriocarcinoma cells, PDE4D was found to be predominantly expressed among all PDE4 isoforms (30). PDE4D inhibitor suppressed migration and invasion of human choriocarcinoma cells *in vitro* via suppressing EMT (30). Conversely, downregulation of PDE4D expression was observed in esophageal cancer tissues, and it was found to bind with lncRNA MANCR promoting esophageal cancer cell proliferation and inhibiting cell apoptosis (102). By interacting with other proteins and signaling pathways, cAMP effectively regulates the status of diverse cell types, including HCC (103). Decreased intracellular levels or inhibition of PDE4D hinders the proliferation of HCC cells, exhibiting tumor-suppressive properties (104). Consistent with this finding, our previous research has established that PDE4D is overexpressed in HCC tissues and synergistically promotes tumor progression with YAP (105).

Concurrently, PDE4D has been reported to impact the therapeutic efficacy of drugs. By genome-wide screening, elevated levels of PDE4D correlated with diminished responsiveness to docetaxel (TXT) therapy, whereas reduced expression enhanced the efficacy of TXT (106) in hypopharyngeal cancers. Conversely, low PDE4D expression was associated with poor outcomes in bladder cancer patients (107). Furthermore, the downregulation of intracellular cAMP through PDE4D enhanced the efficacy of IFN-α against bladder cancer in both *in vitro* and *in vivo (* 107). Interestingly, study revealed that roflumilast-induced upregulation of PDE4D expression works in concert with IFN-α, and amplifying the antiproliferative action of IFN-α on bladder cancer cells *in vitro* and vivo (107). Relative to all other PDE4 subtypes, PDE4D exhibits significantly higher expression in metastatic Caki-1 cells (108). PDE4D knockout augmented the sorafenib-induced apoptosis in Caki-1 cells. These studies highlight the substantial potential of PDE4D in the efficacy of anticancer therapies, necessitating further research to unravel its exact role.

## 3 PDE4 and drug resistance

Drug resistance is the main cause of tumor treatment failure and recurrence. The causes of tumor cell resistance are multiple, including reduced intracellular drug accumulation (109), increased drug efflux (109), alterations in DNA repair mechanisms (110), cancer stem cells (CSC) (111), and modifications in cell death pathways (112). PDE4 subtypes are abnormally expressed in cancer stem cells from various tumors, including breast cancer (111), glioma (37, 113), prostate cancer (114), and leukemia (115). For example, the expression of the PDE4A1 protein is increased in breast CSCs with reduced levels of cAMP (111). The combination of rolipram with paclitaxel demonstrated a synergistic effect and effectively inhibiting the growth of CSCs. Tamoxifen is widely used as the standard first-line adjuvant therapy for estrogen receptor (ER)-positive breast cancer patients, particularly in premenopausal women (116). These studies have demonstrated that tamoxifen administered as adjuvant therapy for at least five years reduces the recurrence rate and mortality of breast cancer by roughly half and one-third, respectively (117). Nevertheless, despite its clinical successes, tamoxifen encounters a significant challenge in that approximately 20%-30% of high-risk, late-stage ER-positive breast cancer patients develop either *de novo* or acquired resistance to the drug (33, 118). The mechanism of tamoxifen resistance is partially understood. By whole-transcriptome sequencing, PDE4D was identified as a potential mediator of tamoxifen resistance (33) (**Figure 5**). Moreover, an elevation in PDE4D expression has been observed in tamoxifen-resistant cells and breast cancer tissues. Resistance to tamoxifen treatment was reversed by PDE4D siRNA or cAMP analogs, which restore sensitivity by activating JNK and P38 pathways and concurrently suppressing AKT activation. Interestingly, aspirin was identified to influence cAMP levels both *in vitro* and *in vivo* by modulating PDE4D activity, and overcoming tamoxifen resistance (33). Acquired resistance to standard-of-care (SOC) treatments for estrogen receptor-positive (ER+) breast cancer, including endocrine therapy and CDK4/6 inhibitors, significantly diminishes patient survival (119, 120). In non-resistant cells, SOC treatment downregulates PDE4D, resulting in elevated cAMP levels, which prompts PKA-dependent phosphorylation of mitochondrial COXIV-I, the generation of reactive oxygen species (ROS), and DNA damage (121). However, during the development of SOC resistance, an ER-to-EGFR switch activates c-Jun, which in turn induces overexpression of PDE4D. Of note, combining SOC with inhibitors of PDE4D, EGFR, or PARP1 has been shown to overcome SOC resistance (121) (**Figure 5**). As a rare and highly aggressive form, inflammatory breast cancer (IBC) presents distinctive clinical features with a low survival rate (122). At the same time, PDE4A exhibited a special role in paclitaxel-resistant IBC cells (123). An increase in AMP, cAMP, and PDE4A expression is observed in paclitaxel-resistant cells, suggesting a potential upregulation of the entire cAMP pathway (123). However, the knockdown of PDE4A has a minimal impact on paclitaxel sensitivity, which may be attributed to the activation of other PDE4 subtypes. Further investigation revealed that pSTAT3 regulates the expression of genes associated with inflammation, EMT, and PDE4A in resistant cells, contributing to paclitaxel therapeutic resistance.

Studies have found that miR-494 is significantly downregulated in gastric cancer cells compared to normal gastric epithelial cells. In addition, its expression is reduced in doxorubicin-resistant gastric cancer cells (AGS/dox) compared to parental cells (32). The increased expression of miR-494 inhibits the expression of PDE4D mRNA and protein in gastric cancer cells. Luciferase assays show that miR-494 directly targets the 3’ untranslated region (3’UTR) of PDE4D. Additionally, restoration of PDE4D reverse the drug sensitivity of in gastric cancer cells with miR-494 overexpression (32). Studies have confirmed that PDE4D is the predominant subtype of PDE4 in either normal or neoplastic renal cells (108). Knockdown of PDE4D or treatment with PDE4 inhibitors enhances the inhibition of sorafenib (108). Similarly, PDE4 inhibitors have also promising synergistic anti-tumor effects in B-cell lymphoma (124, 125).

## 4 PDE4 and tumor immunotherapy

Cancer immunotherapy represents a revolutionary approach in cancer (126, 127). However, despite remarkable progress and groundbreaking successes, especially with checkpoint inhibitors (128–130) and CAR T-cell therapies (131–133), not all patients respond equally, and many patients become resistant to them. PD-L1 (Programmed Death-Ligand 1) serves as a target for immune checkpoint inhibition in cancer therapy, preventing cancer cells from evading immune system attacks by binding to the PD-1 receptor on T cells. Given the role of PD-L1 in suppressing anti-tumor T cell activity, considerable efforts have been made to identify regulators of PD-L1 expression. In immune system cells, the PDE4 family is responsible for a major portion of cAMP hydrolysis (134). In DLBCL cells, the cAMP effectors, specifically PKA and CREB, stimulate the transcription and secretion of IL-10, IL-8, and IL-6. This initiates an autocrine feedback loop, which in turn activates the JAK/STAT signaling pathway, ultimately resulting in elevated expression of PD-L1 on the cell surface (35). Among the members of the PDE4 family, PDE4B stands out as a pivotal regulator of cAMP levels in lymphocytes (135, 136). In PDE4B knockout mice, the proportion of B cells and T cells expressing PD-L1 is significantly higher than in wild-type mice (35). Crucially, despite its broad immunosuppressive properties, the PDE4 inhibitor roflumilast did not diminish the clinical activity of checkpoint inhibitors in a B-cell lymphoma mouse model. Indeed, PDE4 inhibition led to the emergence of a favorable anti-tumor immune profile, including a relative increase in CD8+ cytotoxic T cells (35). Similarly, the PDE4/cAMP pathway may also play a crucial role in regulating PD-L1 and immune infiltration in LUAD (137). Tumor mutation burden (TMB), defined as the number of somatic mutations in a tumor genome after excluding germline mutations, is widely regarded as a potential predictor of immune responsiveness. In a study, bioinformatic screening of LUAD cases with varying TMB values identified PDE4D as a gene associated with the efficacy of immunotherapy (34). High expression of PDE4D correlates with reduced infiltration of CD8+ T cells, suggesting a relative inhibition of the tumor immune microenvironment in LUAD. Moreover, increased PDE4D expression in LUAD patients who were insensitive to PD-1 therapy was consistent with reduced infiltration of CD8+ T cells. Mechanistically, PDE4D negatively regulates the expression and secretion of IL-23 in LUAD cells through cAMP. IL-23 derived from tumor cells fosters a self-amplifying loop of IL-9 secretion through an NF-κB-dependent mechanism, thereby enhancing the cytotoxic function of cytotoxic T lymphocytes (34) (**Figure 5**). These research findings may help to inform the clinical application of FDA-approved PDE4 inhibitors.

Glioblastoma (GBM) is often referred to as “immune cold” characterized by low T cell infiltration. This is not only attributed to the low expression of tumor antigens and a highly immunosuppressive tumor microenvironment but also to the tightly regulated blood-brain barrier that hinders the infiltration of immune cells into the tumor (138). Endothelial cells derived from glioblastomas (GdECs) are thought to arise from glioblastoma stem cells (GSCs) and participate in the formation of vasculogenic mimicry, which facilitates the growth and invasion of cancer (139). Through a rigorous drug screening approach, two cAMP activators have been identified as effectively inhibiting tubule structure formation in human GSCs (140). Indeed, combining cAMP activators with PD-1 blockade therapy resulted in a significant extension of survival in mice, accompanied by the observation of a robust memory immune response (141). Unstable responses to bevacizumab pose a major challenge in the antiangiogenic treatment of high-grade gliomas, which appears to be associated with increased levels of HIF1α and activation of AKT within tumors following treatment (142, 143). Studies show that there is an interaction between PDE4A and HIF1α in tumor cells (58). Selectively inhibiting PDE4 not only blocks this interaction but also suppresses angiogenesis and enhances the efficacy of bevacizumab in an orthotopic glioma model, opening new directions for anti-angiogenic treatment in malignant gliomas (36).

## 5 PDE4 inhibitors in cancer

Rolipram and Ro 20-1724 were first identified as high-selectivity PDE4 inhibitors (144). Subsequently, many specific PDE4 inhibitors have been found and are considered anti-inflammatory agents for asthma and COPD, as they can reduce oxidative stress, TNF-α production, and cytokine generation. Roflumilast, marketed as Daxas^®^, was the first PDE4 inhibitor developed for the treatment of COPD (145). Additional indications are also being explored for roflumilast. Phase IV studies have shown that roflumilast reduce fat mass, leading to weight loss in women with obese polycystic ovary syndrome (PCOS); However, these reductions are less pronounced than those induced by liraglutide (146, 147). Roflumilast has also been tested for its potential to enhance cognition and information processing abilities in healthy individuals, with promising results observed (148). Apremilast, a third-generation PDE4 inhibitor, was approved in 2014 for the treatment of adult patients with psoriatic arthritis (PsA) and moderate-to-severe plaque psoriasis. A significant amount of preclinical data has validated the potential of PDE4 inhibitors as therapeutic agents in the treatment of schizophrenia and cognitive dysfunctions (149–152). Based on preclinical findings, the novel PDE4 inhibitor ASP9831 was tested in Phase I and Phase II trials for nonalcoholic steatohepatitis (NASH), but failed to improve biochemical biomarkers of the disease (153).

Furthermore, a multitude of preclinical and clinical investigations has substantiated the critical role of PDE4 inhibitors in cancer treatments (**Table 2**). Preclinical studies and clinical data collectively indicate that roflumilast demonstrate antitumor activity in B cell lymphomas (40, 125). In conjunction with cAMP-enhancing agents, the PDE4 inhibitor rolipram has exhibited the ability to inhibit triple-negative breast cancer *in vitro* and vivo (41). Likewise, apremilast prompted tumor regression in murine models of colorectal cancer (42). Possibly even more significantly, precise inhibition of PDE4D, whether through genetic approaches or by employing the PDE4D inhibitor Gebr-7b, re-established chemosensitivity in estrogen receptor-positive breast cancer cells that had become resistant to chemotherapy (33). Furthermore, studies have shown that rolipram enhance the therapeutic effect of bevacizumab on gliomas *in vitro (* 36, 37). A clinical case report describes the use of apremilast in managing pustular psoriasis that occurred concurrently in a patient with Ewing’s sarcoma, following chemotherapy with Ifosfamide and Etoposide (154). This suggests that apremilast may offer a novel therapeutic strategy, particularly in the management of cutaneous side effects associated with chemotherapy.

**Table 2:** PDE4 inhibitors and cancers.

PDE4 inhibitors	Cancers	Effects
Apremilast	Colorectal cancer (42)	Induced HKe3 cells apoptosis in a three-dimensional culture by inducing caspade-1 expression
Aspirin	Breast cancer (33)	Reversal of tamoxifen resistance by inhibiting PDE4D/cAMP/ER stress/p38-JNK signaling
Cilomilast	Prostate cancer (155)	Decreased wet weight and increased apoptosis *in vivo* and *vitro*.
Curcumin	Human umbilical vein endothelial cells (156)	Anti-angiogenic and anti-tumor proliferation
DC-TA-46	HepG2 Cells	Caused a marked increase of intracellular cAMP level and a dose- and time-dependent effect on cell growth
Eggmanone	Prostate cancer (114)	Overcome cell chemoresistance via PDE4D inhibition
GEBR-7b	Colorectal cancer (28)	Suppressed the proliferation of CRC-GNASmt cells
Idelalisib	Diffuse large B-cell lymphoma and cell (124)	Suppressing tumor growth and PI3K activity
NCS 613	Lung cancer (157)	Anti-inflammatory and anti-proliferation
NEO214	Glioblastoma (158, 159)	Overcoming chemoresistance by autophagy inhibition
	Multiple myeloma (160)	As an inducer of endoplasmic reticulum
Ro 20-1724	Colorec tal cancer (28)	Suppressed the proliferation of GNASmt cells without an effect on parental cells
Roflumilast	Pancreatic cancer (29)	Inhibited STK11-KO cell migration
	Clear cell renal cell carcinoma (108)	Enhances anti-tumor effects of sorafenib and attenuates MAPK/ERK
	B-cell lymphoma (35, 75, 124, 125, 161)	Inhibited the expression of PDE4b, VEGF secretion and cell proliferation
	Prostate cancer (162)	enhances cisplatin cytotoxicity and protect from cisplatin-induced testicular toxicity
	Ovarian cancer (163)	Up-regulated the expression of FtMt in ovarian cancer via cAMP/PKA/CREB signals
	Lung cancer (58, 164)	Inhibited benzo(a)pyrene-induced murine lung cancer model
	Hepatocellular carcinoma (105)	Disturbed the interaction of PDE4D and YAP
Rolipram	Breast cancer (111, 165)	Combination with paclitaxel indicated synergistic consequences
	Human choriocarcinoma cells (30)	Suppressed migration and invasion by inhibiting epithelial-mesenchymal transition
	Glioblastoma (26, 36, 37, 53, 113, 166, 167)	Induced apoptosis by effecting the PKA and Epac1/Rap1 and MMPK pathways
	Hepatocellular carcinoma (168)	Affected HepG2 Cell Cycle
	Lymphocytic leukemia (169, 170)	Inhibited the IFN regulatory factor 5 and NF-kB p65 nuclear translocation
	Colorectal cancer (82)	Inhibited the cell proliferation by suppressing PDE4B
	Melanoma (171)	Inhibited PDE4B and PDE4D mRNAs and cell growth
Zardaverine	Lung cancer (137)	Decrease PD-L1 expression
Zl-n-91	Acute myeloid leukemia (172)	Inhibited cell growth by attenuating mitochondrial function through a Wnt/β-catenin pathway
	Triple-negative breast cancer (173)	Increased Bax level and reduced Bcl-2 expression and downregulation of the cell cycle-related proteins

In pharmacotherapy, a critical challenge lies in determining the minimum effective dose, which ensures therapeutic efficacy while minimizing drug utilization as much as possible. In other words, a central issue in drug development and clinical practice is how to administer the smallest dose of medication while maintaining its therapeutic effect. It was observed that in MCF-7 cells, which are responsive to hormonal stimuli, chylomicrons demonstrated superior performance over liposomes. Additionally, when rolipram was encapsulated within these vesicles, its IC50 value decreased fourfold relative to the administration of free rolipram (165). Conversely, the IC50 value for Rolipram encapsulated in liposomes was notably higher (165). The underlying mechanisms behind these observations can potentially be better understood through detailed molecular docking investigation. This provides significant guidance for achieving more effective drug delivery.

However, the clinical use of PDE4 inhibitors has faced challenges due to their associated side effects. For instance, although rolipram holds potential pharmacological effects, it has a considerably narrow therapeutic window. In clinical trials, adverse reactions such as nausea, vomiting, and headaches frequently occurred. These adverse effects of rolipram have significantly limited its clinical application (174). While roflumilast demonstrated superior performance to rolipram in clinical trials, gastrointestinal adverse reactions still occurred in 9.5% of cases, characterized by symptoms such as diarrhea, nausea, headache, weight loss, urinary tract infections, and psychiatric disorders (175). Nevertheless, patients may experience adverse reactions such as weight loss (10%), nausea (8.9%), diarrhea (7.7%), nasopharyngitis (2.6%), and upper respiratory tract infection (3.9%) (175). Several hypotheses have been proposed regarding the mechanisms behind PDE4-induced side effects (such as hypothermia, dizziness, diarrhea, nausea, vomiting, etc.), including subclass selectivity, partial inhibition, subcellular compartmentalization, and tissue-specific distribution, but none of these hypotheses has been fully substantiated (176). To overcome the adverse effects induced by PDE4 inhibitors, there is a need for more refined target specificity and a deeper exploration of PDE4 subtype particularities. This involves identifying and leveraging those subtypes that do not contribute to adverse reactions as new targets for drug development. It will require researchers to intricately decipher the functional differences between subtypes at the molecular level and to develop next-generation PDE4 inhibitors that can precisely act on the subtypes relevant to therapy without affecting others that may be implicated in side effects.

## 6 Conclusion

Emerging evidences shed light on the role of PDE4 and PDE4 inhibitors in cancer. However, the exact function of PDE4 isoforms and inhibitors in tumor development, drug resistance and immunotherapy remain debatable. Because of the ubiquitous distribution of PDE4, this superfamily of enzymes plays a role in a wide variety of biological processes. Nevertheless, the biological roles played by specific PDE4 isozymes vary due to their unique expression patterns at the level of tissues/organs, cell types and subcellular compartments. PDE4 are not only responsible for regulating the total cellular levels of cyclic nucleotides; instead, they generate distinct pockets or nanodomains of cyclic nucleotide signaling. This subcellular compartmentalization of cyclic nucleotide signaling allows a single cell to selectively respond to various extracellular and intracellular signals (15). In addition, different PDE4 inhibitors have different inhibitory effects on PDE4 subtypes, which leads to different effects of PDE4 inhibitors in the treatment of different tumors. For example, zardaverine has shown inhibitory effects on HCC both *in vivo* and *in vitro* (177). However, the inhibitory effect of zardaverine on cells appears to be cell-specific, as zardaverine is just not sensitive to cells, including HepG2 cells. More interestingly, the PDE4 inhibitor rolipram failed to mimic this effect, although it also elevated intracellular cAMP levels. Notably, the PDE4 inhibitor rolipram did not have a significant growth inhibitory effect on the zardaverine-sensitive HCC cell line SMMC-7721, which has a relatively low level of PDE4D expression. Consistent with our findings (105), roflumilast inhibited the growth of HepG2 that highly expressed PDE4D. Thus, these HCC cell lines may have different levels of PDE4D expression, and PDE4D may be responsible for cell proliferation during HCC pathology. Again, a similar situation may exist in other tumors. Alternatively, the regulation of the cAMP pathway by PDE4 inhibitors may depend on cell type and conditions. PDE4 inhibitors have the ability to induce the expression of PDE4 isoforms within endothelial and human airway epithelial cells (178, 179). These findings reinforce the notion that specific PDE4 isoforms are segregated by distinct signalosome complexes that regulate local cAMP signaling and confer distinct functional roles.

In addition, the effect of PDE4 inhibitors on tumor still needs more research to confirm. In the future, therapeutic strategies involving PDE4 inhibitors will place a higher value on subtype selectivity and tissue specificity to maximize anti-tumor effects while minimizing side effects. Identifying the patient populations most likely to benefit from PDE4-targeted therapy through precision medicine approaches is critical to improving treatment outcome. Furthermore, combining PDE4 inhibitors with chemotherapy, targeted therapy or immunotherapy will be the focus of research to inhibit tumor growth and restore and enhance the body’s anti-tumor immune response via multiple pathways.

As research progresses and technology advances, our understanding of the functional roles and molecular interactions of each PDE4 isoform and how their function and/or localization is altered in specific diseases grows, it will become more promising to see how precisely we can target PDE4 isoforms to achieve therapeutic efficacy while minimizing side effects. Also, the refinement of these studies is an important basis for the use of PDE4 as a therapeutic target for tumors.
